# Case 4 – A 79-Year-Old Man with Congestive Heart Failure Due to Restrictive Cardiomyopathy

**DOI:** 10.5935/abc.20150135

**Published:** 2015-10

**Authors:** Sumaia Mustafa, Alice Tatsuko Yamada, Fabio Mitsuo Lima, Valdemir Melechco Carvalho, Vera Demarchi Aiello, Jussara Bianchi Castelli

**Affiliations:** 1 Instituto do Coração (InCor) HC-FMUSP, São Paulo, SP – Brasil; 2 Grupo Fleury Medicina e Saúde, São Paulo, SP – Brasil

**Keywords:** Heart Failure, Cardiomyopathy, Restrictive, Ascites, Cardiomegaly, Heart Arrest

JAP, a 79-year-old male and retired metalworker, born in Várzea Alegre (Ceará, Brazil) and residing in São Paulo was admitted to the hospital in October 2013 due to decompensated heart failure.

The patient was referred 1 year before to InCor with a history of progressive dyspnea triggered by less than ordinary activities, lower-extremity edema, and abdominal enlargement. He sought medical care due to the abdominal enlargement, which was diagnosed as an ascites. He denied chest pain, hospitalization due to myocardial infarction or stroke, hypertension, dyslipidemia, and diabetes.

The patient was a previous smoker and had stopped smoking at the age of 37 years. He was also an alcoholic and reported drinking alcohol for the last time 1 year before.

He was referred to InCor for treatment of heart failure.

An echocardiogram revealed an increased thickness in the septum (17 mm) and free left ventricular wall (15 mm), and a left ventricular ejection fraction of 26%.

The patient reported daily use of enalapril 10 mg, spironolactone 25 mg, furosemide 80 mg, omeprazole 40 mg, and ferrous sulfate (40 mg Fe) three tablets.

On March 12, 2013, his physical examination showed a weight of 55 kg, height of 1.75 m, body mass index (BMI) of 18 kg/m^2^, heart rate of 60 bpm, blood pressure of 90 X 50 mm Hg, and the presence of a hepatojugular reflux. There were no signs of jugular venous hypertension, and the pulmonary and cardiac auscultations were normal. He had ascites, and his liver was palpable 5 cm below the right costal margin. Peripheral pulses were palpable, and a ++/4+ edema was observed.

An ECG (February 23, 2012) had shown a sinus rhythm, heart rate of 52 bpm, PR interval of 192 ms, QRS duration of 106 ms, indirect signs of right atrial overload (wide variability in QRS amplitude between V1 and V2), and left atrial overload (prolonged and notched P waves), low QRS voltage in the frontal plane with an indeterminate axis, an electrically inactive area in the anteroseptal region and secondary changes in ventricular repolarization (Figure 1).

**Figure 1:**
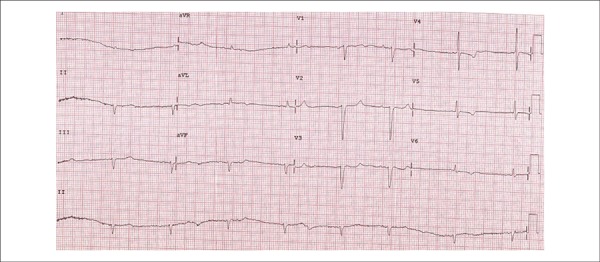
ECG: sinus bradycardia, low-voltage QRS complexes in the frontal plane, indirect signs of right atrial overload (small QRS complexes in V1 and wide QRS complexes in V2), left atrial overload, electrically inactive area in the anteroseptal region.

A chest x-ray showed cardiomegaly.

Laboratory tests performed on April 20, 2012, had shown the following results: hemoglobin 13.1 g/dL, hematocrit 40%, mean corpuscular volume (MCV) 87 fL, leukocytes 9,230/mm^3^ (banded neutrophils 1%, segmented neutrophils 35%, eosinophils 20%, basophils 1%, lymphocytes 33%, and monocytes 10%), platelets 222,000 /mm^3^, cholesterol 207 mg/dL, HDL-cholesterol 54 mg/dL, LDL-cholesterol 138 mg/dL, triglycerides 77 mg/dL, creatine phosphokinase (CPK) 77 U/L, blood glucose 88 mg/dL, urea 80 mg/dL, creatinine 1.2 mg/dL (glomerular filtration rate ≥ 60 mL/min/1.73 m^2^), sodium 131 mEq/L, potassium 6.3 mEq/L, aspartate aminotransferase (AST) 22 U/L, alanine aminotransferase (ALT) 34 U/L, uric acid 6.3 mg/dL, TSH 1.24 µUI/mL, free T4 1.36 ng/dL, prostate-specific antigen (PSA) 1.24 ng/mL. On urinalysis, urine specific gravity was 1.007, pH 5.5, the sediment was normal, and there were no abnormal elements.

A new echocardiographic assessment on April 20, 2012, had shown an aortic diameter of 32 mm, left atrium of 52 mm, septal and posterior left ventricular wall thickness of 15 mm, diastolic/systolic left ventricular diameters of 46/40 mm, and left ventricular ejection fraction of 28%. Both ventricles had diffuse and marked hypokinesia. The valves were normal and the pulmonary artery systolic pressure was estimated at 32 mmHg (Figure 2).

**Figure 2:**
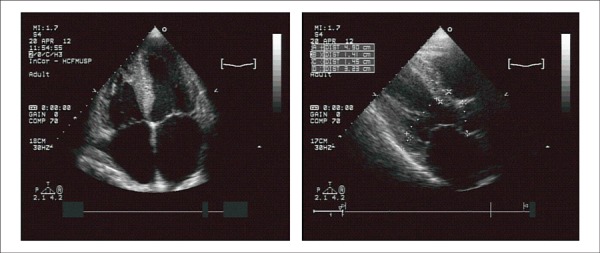
Echocardiogram - a) Four-chamber view: marked enlargement of the left and right atria; b) parasternal long-axis view: enlarged left atrium, left ventricular wall thickening, normal cavity.

A 24-hour electrocardiographic (Holter) monitoring on April 19, 2012, showed a baseline sinus rhythm with a lowest rate of 46 bpm and greatest rate of 97 bpm; 48 isolated, polymorphic, and paired ventricular extrasystoles; 137 atrial extrasystoles; and an episode of atrial tachycardia over three beats with a frequency of 98 bpm. There were no atrioventricular or intraventricular blocks interfering with the conduction of the stimulus.

The patient was transferred from the pacemaker clinic to the general cardiopathy clinic.

During a clinic appointment on January 22, 2013, the patient was asymptomatic and reported the use of enalapril 10 mg, spironolactone 25 mg, furosemide 60 mg, and carvedilol 12.5 mg. His physical examination was normal.

The main diagnostic hypotheses were hypertrophic or restrictive cardiomyopathy.

A testicular ultrasound (September 09, 2013) was normal, except for cystic formations in the right inguinal canal. An abdominal ultrasonography (September 10, 2013) showed substantial ascites and hepatic cysts with internal septations, and no signs of portal hypertension.

After presenting an increase in dyspnea with the development of paroxysmal nocturnal dyspnea, worsening ascites and lower-extremity edema, and paresthesia on hands and feet, the patient was admitted to the hospital.

On physical examination (October 19, 2013) he was oriented and eupneic, with a heart rate of 69 bpm, blood pressure of 80 X 60 mmHg, a normal pulmonary auscultation, cardiac auscultation with arrhythmia and no murmurs, substantial ascites, and edema and hyperemia of the lower extremities.

A chest x-ray (October 21, 2013) showed cardiomegaly and interstitial lung infiltrates; the lateral incidence showed the right ventricle markedly enlarged (Figures 3 and 4).

**Figure 3:**
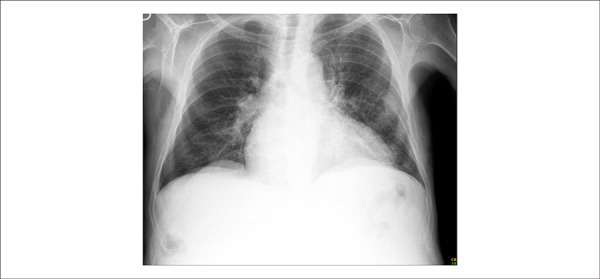
Chest x-ray (October 21, 2013), posteroanterior (PA) view: pulmonary interstitial infiltrates and cardiomegaly.

**Figure 4:**
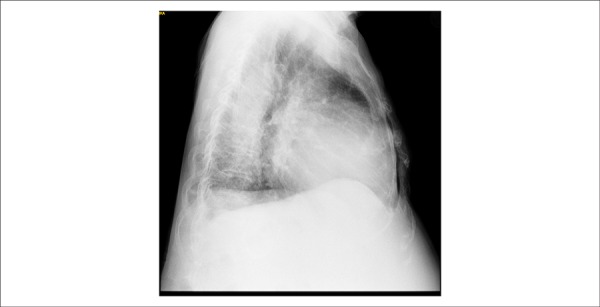
Chest x-ray (October 21, 2013) in lateral view: right ventricle markedly enlarged.

On ECG, the patient presented atrial flutter with variable atrioventricular block, indirect signs of right atrial overload (Peñaloza-Tranchesi sign), heart rate of 61 bpm, low QRS voltage in the frontal plane, intraventricular conduction impairment, left ventricular overload, and secondary changes in ventricular repolarization (Figure 5).

**Figure 5:**
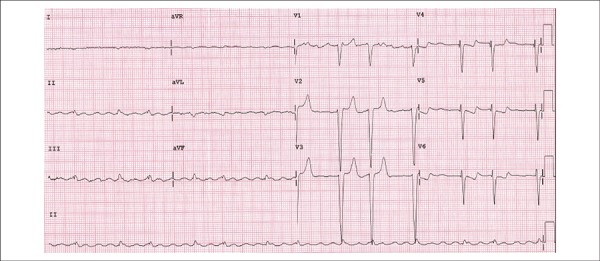
ECG: Atrial flutter, impaired intraventricular conduction, left ventricular overload.

Laboratory tests on October 19, 2013, showed the following results: hemoglobin 13.5 g/dL, hematocrit 42%, leukocytes 7,230/mm^3^ (neutrophils 66%, eosinophils 12%, lymphocytes 13%, monocytes 9%), platelets 232,000 /mm^3^, urea 193 mg/dL, creatinine 2.03 m/dL (glomerular filtration rate of 34 mL/min/1,73 m^2^), sodium 133 mEq/L, potassium 3.9 mEq/L, C-reactive protein (CRP) 18.1 mg/L, vitamin B12 360 pg/mL, folic acid 8.35 ng/mL, total bilirubin 0.75 mg/dL, direct bilirubin 0.37 mg/dL, AST 24 U/L, ALT 16 U/L, gamma-glutamyl transferase (gamma GT) 241 U/L, alkaline phosphatase 166 U/L, iron 71 µg/dL, ferritin 62.9 ng/mL, prothrombin time (PT, INR) 0.95, activated partial thromboplastin time (aPTT, rel) 0.95, ionic calcium 1.09 mmol/L, chloride 89 mEq/L, and arterial lactate 15 mg/dL. Urinalysis showed urine specific gravity of 1.020, pH 5.5, proteinuria 0.25 g/L, epithelial cells 4,000/mL, leukocytes 2,000/mL, erythrocytes 3,000/mL, and hyaline casts 27,250/mL.

Another echocardiogram performed on October 21, 2013, showed a left atrial diameter of 56 mm, septal thickness of 18 mm, posterior wall thickness of 13 mm, left ventricle (diastole/systole) with 46/40 mm, left ventricular ejection fraction of 28%, pulmonary artery systolic pressure estimated at 45 mmHg, marked left ventricular and moderate right ventricular dysfunction, and moderate tricuspid insufficiency.

An ultrasound of the kidneys and urinary tract (October 24, 2013) showed that the left kidney measured 9.6 cm, and the right kidney measured 9 cm and had simple cortical cysts.

Serum protein electrophoresis was normal, and a urinary electrophoresis did not detect proteins. Measurement of serum beta 2-microglobulin was 7 mg/mL (limit for individuals above the age of 60 years = 2.6 mg/mL).

A biopsy of the cheek mucosa (October 23, 2013) showed deposits of amyloid substance in the deep chorion and in the adjacent adipose tissue.

Stool microscopy (October 25, 2013) was positive for *Blastocystis hominis* and *Entamoeba coli*.

A paracentesis drained 3,500 mL of a yellowish fluid with normal cellularity.

During hospitalization, the patient received daily intravenous furosemide 60 mg, carvedilol 25 mg, hydrochlorothiazide 100 mg, hydralazine 75 mg, isosorbide 80 mg, aspirin 100 mg, spironolactone 25 mg, and enoxaparin 40 mg. The patient also received oxacillin 2 g/day for 7 days initially, and later vancomycin, meropenem and teicoplanin, and piperacillin/tazobactam.

A new chest x-ray (November 08, 2013) showed cardiomegaly and an interstitial pulmonary infiltrate suggestive of pulmonary congestion (Figure 6).

**Figure 6:**
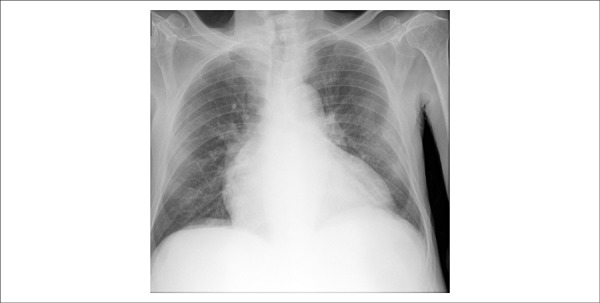
Chest x-ray (November 08, 2013): pulmonary interstitial infiltrates suggestive of pulmonary congestion and cardiomegaly.

During a new paracentesis (November 11, 2013), the aspirated fluid was bloody, and the patient presented hypotension and decreased consciousness, progressing to cardiac arrest with pulseless electrical activity, which was reverted. This was followed by ventricular tachycardia, cardioverted with 200 J.

New tests (November 11, 2013 - morning) showed the following results: hemoglobin 11.9 g/dL, hematocrit 36%, leukocytes 7,780/mm^3^ (neutrophils 83%, eosinophils 2%, lymphocytes 9%, and monocytes 6%), platelets 188,000 /mm^3^, urea 301 mg/dL, creatinine 4.14 mg/dL, sodium 125 mEq/L, potassium 4.4 mEq/L, CRP 97.06 mg/L. On venous blood gas analysis, pH was 7.33, bicarbonate 19.9 mmol/L, and base excess (-) 5.4 mmol/L. Additional tests performed on the same day (November 11, 2013 – 5:44 pm) showed hemoglobin of 6.3 g/dL, sodium of 123 mEq/L, potassium of 5.4 mEq/L, venous lactate of 93 mg/dL, PT (INR) of 3.2 and aPTT (rel) of 1.98.

Later during the day, the patient progressed with shock refractory to high doses of dobutamine (20 µg/kg/min ) and norepinephrine (1.2 µg/kg/min), followed by cardiac arrest with pulseless electrical activity that recovered but was followed by a new irreversible cardiac arrest with pulseless electrical activity during intra-aortic balloon placement (November 11, 2013 – 6:30 pm).

## Clinical Aspects

The patient JAB, a 79-year-old previous smoker and alcoholic man residing in São Paulo, attended an outpatient clinic at InCor due to heart failure which worsened progressively since 2012, requiring hospitalization for treatment.

Heart failure is a systemic and complex clinical syndrome, defined as a cardiac dysfunction that causes the blood supply to be insufficient to meet tissular metabolic demands, in the presence of a normal venous return, or which only meets the demands with high filling pressure^1^.

Prevalence studies estimate that 23 million individuals worldwide have heart failure and that 2 million new cases are diagnosed annually. According to DATASUS information, Brazil has about 2 million individuals with heart failure and 240,000 new cases diagnosed annually^2^.

The main causes of heart failure are hypertension, coronary artery disease, Chagas disease, cardiomyopathies, endocrinopathies, toxins, and drugs, among others^1^. The cardinal manifestations of heart failure are dyspnea and fatigue, and may include exercise intolerance, fluid retention, and pulmonary and systemic congestion^3^. The patient in this case presented with progressive dyspnea triggered by less than ordinary activities, lower-extremity edema, and ascites, which characterized him as class III according to the New York Heart Association (NYHA) classification.

On complementary tests, the echocardiogram showed marked left ventricular hypertrophy with some degree of asymmetry, and reduced ejection fraction. Cardiac hypertrophy is often associated with hypertension or hypertrophic cardiomyopathy, but both present with normal or increased ECG voltage. Therefore, the findings of ventricular hypertrophy associated with decreased ECG voltage in the absence of pericardial effusion are exclusive of infiltrative cardiomyopathies, a group of cardiac disorders within the restrictive cardiomyopathies^4^.

Restrictive cardiomyopathy may occur with a wide variety of systemic diseases. Some restrictive cardiomyopathies are rare in clinical practice and may present initially with heart failure. This type of cardiomyopathy is characterized by filling restriction, with reduced diastolic volume in one or both ventricles, normal or close to normal systolic function, and ventricular wall thickening. It may be idiopathic or associated with other diseases, such as amyloidosis, endomyocardial disease with or without eosinophilia, sarcoidosis, and hemochromatosis, among others^5^. In this case, the presence of amyloid deposits in the cheek mucosa biopsy indicated a diagnosis of amyloidosis, and the increase in serum beta-2 microglobulin reflected a worse prognosis^5^.

Amyloidosis is characterized by deposits of amyloid protein in different organs and tissues. These deposits may be responsible for different types of clinical presentation, with a spectrum that ranges from lack of symptoms to sequential organic dysfunction culminating with death^6^.

Cardiac amyloidosis is caused by amyloid deposits around cardiac fibers, and can be identified by a left ventricular wall thickening exceeding 12 mm in the absence of hypertension with at least one of the following characteristics: conduction disorder and low voltage complexes on the ECG, restrictive cardiomyopathy, low cardiac output, isolate atrial involvement (as commonly seen in elderly individuals) or diffuse involvement affecting the ventricles. In the latter situation, it can cause heart failure with a poor prognosis^4,7^.

Our patient, who was not hypertensive, presented low voltage complexes on the ECG, which were more prominent in the frontal plane, an electrically inactive area in the anteroseptal region, and diffuse changes in ventricular repolarization. This pattern can be found in some diseases in addition to infiltrative cardiomyopathies, such as decompensated hypothyroidism, pericardial effusion, chronic obstructive pulmonary disease, and obesity. Other electrocardiographic information, such as the pattern of infarction, can be found with or without obstructive coronary atherosclerotic disease by deposition of substances in the microcirculation and small intramyocardial arteries^8^.

Amyloidosis may be classified as primary, secondary, or hereditary. Primary amyloidosis, in which AL is the primarily involved protein, may be further subdivided into idiopathic (localized forms) or associated with multiple myeloma or other plasma cell dyscrasias (systemic forms)^9^.

Multiple myeloma is a neoplastic disorder of plasma cells that affects individuals with an average age of 70 years at diagnosis. Some characteristics of the patient in this case could suggest multiple myeloma: age, male gender, renal failure, and cylindruria. However, other important clinical parameters were absent, such as hypercalcemia, anemia, and bone disease. Also, the Bence-Jones protein, which is present in up to 75% of the cases, was not detected on urinary electrophoresis^10^.

The secondary type of amyloidosis results from deposits of AA protein and frequently arises as a complication of infectious or inflammatory processes, such as rheumatoid arthritis (the most common cause), tuberculosis, systemic lupus erythematosus, inflammatory bowel disease, syphilis, or even neoplastic diseases. Pro-inflammatory cytokines, which are present in these disorders, stimulate the hepatic production of serum A amyloid^11^.

Finally, the hereditary type of the disease has an autosomal dominant transmission and may involve several types of amyloid proteins, such as the AA protein in some groups of patients with familial Mediterranean fever, and the ATTR protein (derived from the transthyretin or prealbumin) in familial amyloid polyneuropathy^12^.

As for the treatment, measures to control symptoms related to diastolic heart failure, such as volume control, should be implemented. Diuretics and vasodilators should be administered with caution since the cardiac output in these patients is greatly dependent on increased venous pressures. Specific treatment should be directed to the etiology of the amyloidosis^13^.

After an evaluation in the clinic on January, 2013, the patient received medications that are proven to modify the rates of hospitalization and mortality in heart failure with reduced ejection fraction (beta-blockers, angiotensin-converting enzyme inhibitors, aldosterone antagonist), and symptom-relieving agents (diuretics)^14^. The patient was receiving enalapril 10 mg, spironolactone 25 mg, furosemide 60 mg, and carvedilol 12.5 mg.

After 8 months, due to the decompensated heart failure and hypotension, the patient returned to the emergency room and required hospitalization. The use of conventional therapy for heart failure often worsens the progression of amyloidosis. Therefore, cardiac amyloidosis should be suspected when the patient’s clinical condition worsens in response to conventional treatment, particularly in individuals older than 50 years. The therapy is exclusively symptomatic and should not include digitalis, beta-blockers, angiotensin-converting enzyme inhibitors, or calcium channel antagonists, since some studies have shown an increased sensitivity to these drugs which can lead to hypotension and intensification of conduction disorders^15^.

Therefore, the decompensation of the patient’s heart failure with deterioration of the ascites culminated in two paracenteses, with the last paracentesis probably accompanied by a puncture accident due to the appearance of bloody fluid, decrease in red blood count, and hypovolemic shock associated with cardiogenic shock, culminating in a mixed refractory shock and cardiac arrest with pulseless electrical activity **(Dr. Sumaia Mustafa, Dr. Alice Tatsuko Yamada).**

**Diagnostic hypotheses:**
1. Heart failure due to restrictive cardiomyopathy (probably cardiac amyloidosis associated with multiple myeloma);
2. Decompensated heart failure;
3. Cause of death: mixed shock (hypovolemic + cardiogenic) with cardiac arrest with pulseless electrical activity **(Dr. Sumaia Mustafa, Dr. Alice Tatsuko Yamada)**.

### Autopsy

**Figure 7:**
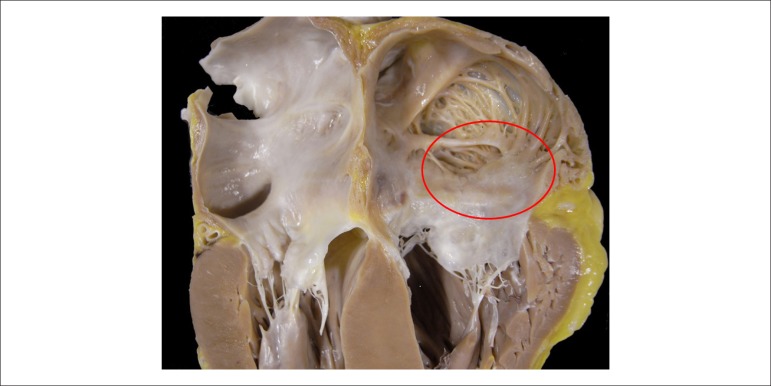
Gross Section of the heart base showing biatrial enlargement and thickening of the cardiac walls. Note the granular aspect of the right atrial endocardium (area demarcated with an ellipse).

The heart weighed 680 g and was increased in volume due to moderate cavity dilation and wall thickening in all four chambers (Figure 7). The myocardium had an increased consistency. The endocardium of the atria, in particular, was finely granular and brown-yellowish in appearance. There were no significant changes in the valves, and the coronary arteries were armed without significant obstruction of their lumen.

**Figure 8:**
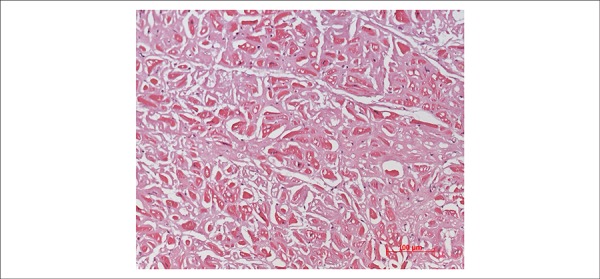
Photomicrography of the ventricular myocardium showing atrophic cardiomyocytes due to deposits of amorphous eosinophilic material in the interstitium. Hematoxylin and eosin staining (20x objective magnification).

**Figure 9:**
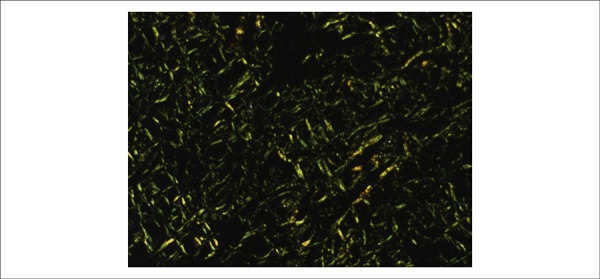
Photomicrography of myocardial tissue obtained under polarized light. Note the greenish material that corresponds to amyloid substance stained by Congo red (5x objective magnification).

**Figure 10:**
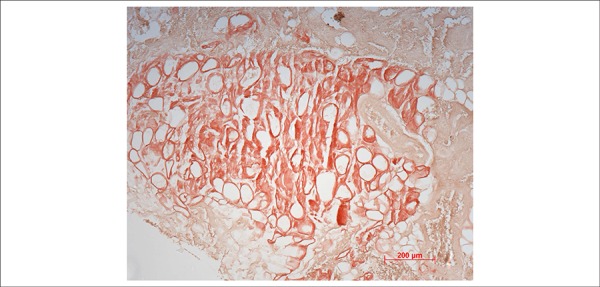
Biopsy of the cheek mucosa performed approximately 1 month before death. Note that the mucosal chorion reacts positively to Congo red (photomicrography obtained under conventional microscopy with a 10x objective magnification).

**Figure 11:**
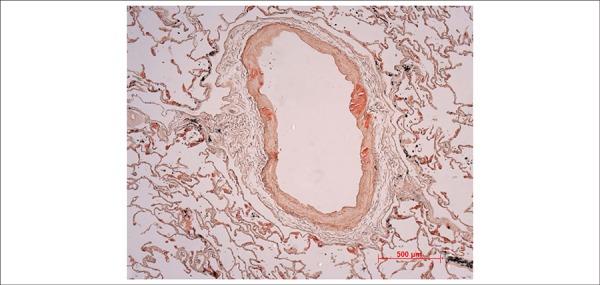
Photomicrography of a peripheral muscular pulmonary artery showing areas of positivity for deposits of amyloid in the arterial wall (Congo red staining photographed under conventional microscopy, 5x objective magnification).

Histological examination of the myocardium showed extracellular deposits of amorphous and eosinophilic material promoting atrophy of the contractile cells. These deposits stained positive with Congo red when observed under polarized light (Figures 8 and 9). This same material was present in the interstitium of the cheek mucosa evaluated by biopsy (Figure 10) according to data from the clinical history. Deposits were also observed in the tunica media of muscular arteries in both lungs (Figure 11) and in the renal hilum.

Bone marrow histological examination showed hypercellularity of moderate degree for the patient's age, and no signs of monoclonal proliferation. Immunohistochemical reactions for immunoglobulin kappa and lambda light chains were inconclusive, and CD138 labeling showed no proliferation of plasma cells.

Autopsy findings included a 4-cm hepatic cyst in the right lobe lined with flat cells without atypia, and retention cysts in the right kidney. The right adrenal weighed 44 g and was increased in volume and completely calcified. The histological examination showed only calcification and was inconclusive for the possibility of prior malignancy.

There was a voluminous serosanguinous ascites and a serous pericardial effusion. We found no visceral or abdominal vessel injury resulting from the paracentesis and the amount of bloody material in the ascitic fluid was small.

Histologically, there were signs of congestive heart failure in the lungs and liver **(Dr. Vera Demarchi Aiello)**.

**Diagnoses:** Cardiovascular amyloidosis;

Congestive heart failure;

Calcified nodule in the right adrenal gland **(Dr. Vera Demarchi Aiello).**

### Mass spectrometry

Mass spectrometry gathers all qualities to establish an unequivocal diagnosis of amyloidosis since it has a high sensitivity and ability to identify the proteins through sequencing^16^. Therefore, we adopted an approach based on shotgun proteomics to identify the amyloid deposits in the sample.

Sections of heart tissue containing amyloid deposits (confirmed by staining with Congo red) fixed in formalin and embedded in paraffin were dissected and the proteins were then extracted with Liquid Tissue® MS Protein Prep Kit (Expression Pathology) according to the manufacturer's protocol. After digestion with trypsin, the resulting peptides were analyzed by high-resolution liquid chromatography-mass spectrometry using the mass spectrometer Q-Exactive (Thermo Fisher Scientific). The acquisitions of spectral data were carried out using the DDA (date dependent analysis) mode with a selection of the 10 most abundant ions for sequencing by HCD (Higher-energy collisional dissociation). The data were processed with the software MaxQuant. The proteomic analysis was performed in triplicate.

The processed data generated lists of proteins representing the protein content of the sample. In total, 25 possible amyloid proteins were investigated in these lists in order to determine the identity of the deposited substance. There were 15 peptides belonging to transthyretin that together covered 76.2% of the full sequence of the protein.

To confirm the result, we also evaluated the abundance of different peptides present in the sample. Among the 25 most abundant peptides, three belonged to transthyretin (ALGISPFHEHAEVVFTANDSGPR, TSESGELHGLTTEEEFVEGIYK, and GSPAINVAVHVFR). The others were assigned to actin, myosin, desmin, and myoglobin, confirming the identity of the amyloid protein **(Dr. Fabio Mitsuo Lima and Dr. Valdemir Melechco Carvalho- Fleury Group).**

## Conclusion

Cardiovascular amyloidosis due to deposition of transthyretin **(Dr. Vera Demarchi Aiello, Dr. Jussara Bianchi Castelli, Dr. Fabio Mitsuo Lima and Dr. Valdemir Melechco Carvalho)**.

### Comments:

This case demonstrates how important it is in amyloidosis to investigate the deposited substance. Amyloidosis is a generic name to describe a group of diseases characterized by extracellular deposits of different substances in different organs. These substances are fibrillar proteins that become insoluble with changes in their spatial conformation. More than 20 types of proteins have been described in these deposits^16^. From an anatomopathological perspective, the deposits can be characterized by immunohistochemical reactions, but with some restrictions as described below. The cardiovascular system is most often affected by the AL protein (deposits of light-chain immunoglobulin), senile, and familial forms^17,18^.

The pathologist may identify neoplastic proliferation of plasmocytes producing the deposited immunoglobulins by bone marrow examination labeled for these cells. In tissue preparations, the pathologist may demonstrate by immunohistochemistry if the deposited substance is one of these immunoglobulins. Some authors recommend a biopsy of other organs before the endomyocardial biopsy to confirm the diagnosis and identify the type of amyloid^19^. In this case, immunohistochemical labeling was not helpful in establishing the diagnosis, because it was inconclusive to the type of protein deposited.

Although there are reports in the literature of identification of transthyretin in tissues by immunohistochemical reactions, this was not possible in this case. However, with mass spectrometry analysis, we identified that the deposited protein was transthyretin, which is usually present in senile and familial forms of amyloidosis. In this patient, the familial form was less likely due to the exclusive involvement of heart and blood vessels. However, only a genetic research and evaluation of other members of the family could exclude it completely.

Another point that deserves discussion in this case is the laboratory report of high levels of immunoglobulin E. We could assume that this referred to the deposited protein, but the diagnostic methods performed to complement the autopsy revealed that this was not the case.

**Dr. Vera Demarchi Aiello and Dr. Jussara Bianchi Castelli (Pathology Laboratory, InCor, FMUSP).**

Heart Failure; Cardiomyopathy, Restrictive; Ascites; Cardiomegaly; Heart Arrest.

**Editor da Seção:** Alfredo José Mansur (ajmansur@incor.usp.br)

**Editores Associados:** Desidério Favarato (dclfavarato@incor.usp.br)

Vera Demarchi Aiello (anpvera@incor.usp.br)
